# Oxygenation Performance of Different Non-Invasive Devices for Treatment of Decompression Illness and Carbon Monoxide Poisoning

**DOI:** 10.3389/fphys.2022.885898

**Published:** 2022-04-26

**Authors:** Andrea Köhler, Felicitas M. Zoll, Thomas Ploner, Alexander Hammer, Michael Joannidis, Herbert Tilg, Armin Finkenstedt, Frank Hartig

**Affiliations:** Division of Intensive Care and Emergency Medicine, Department of Internal Medicine I, Medical University Innsbruck, Innsbruck, Austria

**Keywords:** arterial oxygen partial pressure, oxygen masks, diving accident, PaO2, non-invasive ventilation, carbon monoxide poisoning, decompression illness

## Abstract

**Study Objective:** Application of high concentrations of oxygen to increase oxygen partial pressure (pO2) is the most important treatment for patients with carbon monoxide intoxication or divers with suspected decompression illness. The aim of this study was to evaluate the oxygenation performance of various non-invasive oxygen systems.

**Methods:** The effect of different oxygen systems on arterial pO2, pCO2 and pH and their subjective comfort was evaluated in 30 healthy participants. Eight devices were included: nasal cannula, non-rebreather mask, AirLife Open mask, Flow-Safe II CPAP device, SuperNO_2_VA nasal PAP device, all operated with 15 L/min constant flow oxygen; nasal high-flow (50 L/min flow, 1.0 FiO2), non-invasive positive pressure ventilation (NPPV, 12 PEEP, 4 ASB, 1.0 FiO2) and a standard diving regulator (operated with pure oxygen).

**Results:** Diving regulator, SuperNO_2_VA, nasal high-flow and NPPV achieved mean arterial pO2 concentrations between 538 and 556 mm Hg within 5 minutes. The AirLife Open mask, the nasal cannula and the non-rebreather mask achieved concentrations of 348–451 mm Hg and the Flow-Safe II device 270 mm Hg. Except for the AirLife open mask, pCO2 decreased and pH increased with all devices. The highest pH values were observed with NPPV, diving regulator, Flow-Safe II and nasal high-flow but apparent hyperventilation was uncommon. The AirLife Open and the non-rebreather mask were the most comfortable, the SuperNO_2_VA and the nasal cannula the most uncomfortable devices.

**Conclusion:** A standard diving regulator and the SuperNO_2_VA device were equally effective in providing highest physiologically possible pO2 as compared to nasal high-flow and NPPV.

## Introduction

The application of oxygen with various devices is a common therapeutic strategy in preclinical and clinical emergency medicine. In some emergencies, such as smoke and carbon monoxide (CO) intoxication and decompression illness (DCI), arterial oxygen partial pressures (pO2) far beyond physiological values are needed as life-saving acute treatment (Brubakk and Neumann, 2002; Eichhorn et al., 2018; Juttner et al., 2021).

CO intoxication is a common problem worldwide, either in form of smoke poisoning or in form of a solitary intoxication. The clinical symptoms are highly variable and range from headache over alterations in mental status to coma. Compared to oxygen, CO has a 200–300 times higher affinity to hemoglobin and other heme-containing enzymes like myoglobin and cytochrome-c oxidase (Rose et al., 2017). Hence, inhaled CO displaces oxygen from these enzymes and blocks oxygen transport and the respiratory chain (Weaver, 1999; Weaver, 2009). This results in a variety of complications including tissue hypoxia as well as myocardial and cerebral damage (Weaver, 1999). Tissue damage can be significantly reduced by eliminating CO from heme-containing enzymes as fast as possible. The most important emergency intervention is the immediate application of pure oxygen, very often as a bridging therapy until a hyperbaric oxygen (HBO) therapy can be organized (Juttner et al., 2021). Normobaric administration of 100% oxygen reduces the CO dissociation half-life from 320 min to approx. 74 min (Ernst and Zibrak, 1998; Weaver et al., 2000) HBO therapy at 3 bar reduces carboxyhemoglobin half-life even further to about 23 min (Pace et al., 1950; Peterson and Stewart, 1970; Jay and McKindley, 1997) and can improve the neurological outcome. HBO therapy also increases the physically dissolved oxygen in the blood which may compensate the hypoxia caused by CO-hemoglobin compounds (Weaver, 1999).

DCI often occurs in remote areas, where only limited first aid kits but often oxygen from other divers or paramedics are available. DCI is caused by bubble formation from dissolved inert gas during a compressed gas dive. These bubbles may develop because of supersaturation due to a too fast pressure drop when ascending. Depending on size, number and location, these emboli can cause subclinical to life-threatening damage in all kind of tissues and organ systems (Vann et al., 2011; Hartig et al., 2020). Breathing pure oxygen is the most important and effective therapy, because oxygen not only dissolves the bubbles, but also compensates the hypoxia in affected tissues (Longphre et al., 2007; Mitchell et al., 2018a). Thus, HBO therapy in a pressure chamber with ambient pressures up to 2.8 bar is the standard treatment for DCI (Brubakk and Neumann, 2002).

Both in severe CO intoxication and DCI, HBO therapy should be initiated as soon as possible. Until patients have been transferred to a specialized center where HBO therapy is available, treatment with oxygen should be aimed at achieving the highest possible arterial pO2.

There are numerous oxygen systems on the market, ranging from simple nasal cannulas to non-invasive positive pressure ventilation (NPPV) devices. Various studies investigated the effect of different oxygen systems on blood oxygen saturation or tissue oxygen partial pressure (Vitaliti et al., 2017; Blake et al., 2018). However, the efficacy of different oxygen systems to increase effective arterial pO2 has not been studied yet. Beside efficacy, practicability, transportability and respiratory comfort are also important for the use of oxygen systems in an emergency setting.

The primary aim of this study was to investigate common oxygen systems used in a modern emergency room for their effective oxygenation performance. Furthermore, the systems were compared for their respiratory comfort and practicability. The findings of this study shall improve the treatment of patients with DCI and smoke and CO intoxication by selecting the most appropriate oxygen system for those emergencies.

## Methods

### Study Design and Setting

Eight different non-invasive oxygen systems were tested in 30 healthy participants. The study was approved by the ethics committee of the Medical University Innsbruck (reference 1,148/2021) and the national competent authorities (BASG reference 100045366). Written informed consent was obtained from all participants.

**FIGURE 1:**
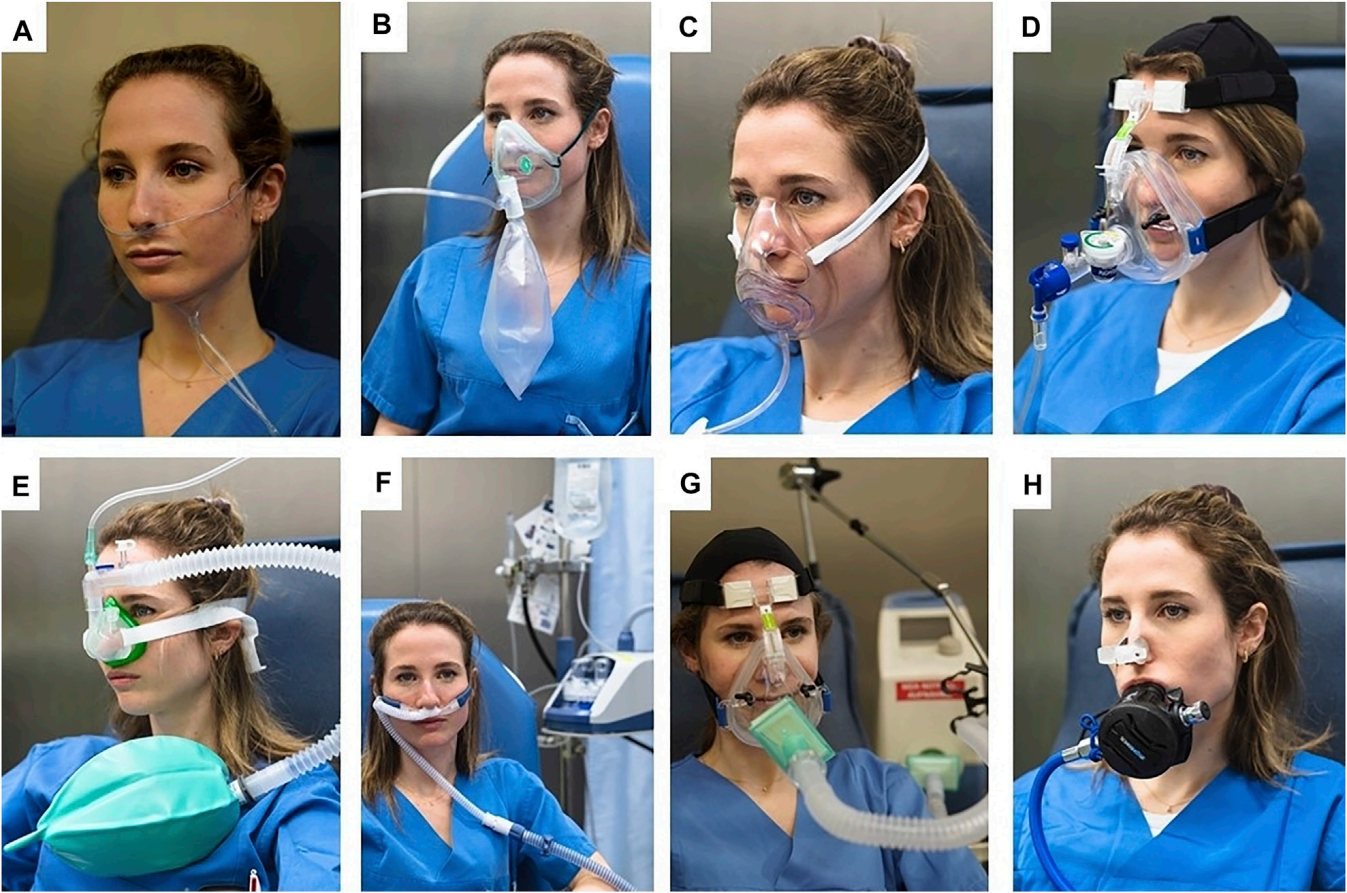
The oxygen devices used in this study **(A)** Standard nasal cannula **(B)** Standard non-rebreather mask with expiration valves. **(C)** Air Life Open. An oxygen mask that allows patients to drink, eat and talk without removing the mask **(D)** Flow-Safe II. A CPAP device that can be used with a standard NPPV face mask and constant flow oxygen. **(E)** SuperNO_2_VA. A nasal PAP device also used with constant flow oxygen **(F)** Nasal high-flow with a Fischer&Paykel Airvo2. **(G)** Non-invasive positive pressure ventilation with a Dräger Carina **(H)** Standard diving regulator. Oxygen was delivered by a scuba tank.

The following systems were tested: A) Standard nasal cannula (Ningbo Shengyurui Medical Appliances Co., Ningbo City, China), B) standard non-rebreather mask with expiration valve (EcoLite 181015, Intersurgical Ltd., Wokingham, United Kingdom), C) AirLife Open oxygen mask (Vyaire Medical, Mettawa, USA), D) Flow-Safe II continuous positive airway pressure device with face mask (Mercury Medical, Clearwater, USA), E) SuperNO_2_VA nasal positive airway pressure (PAP) ventilation device (Vyaire Medical, Mettawa, USA), F) nasal high-flow using an Airvo2 humidifier and flow generator (Fischer&Paykel, Auckland, New Zealand), G) non-invasive positive pressure ventilation using a Dräger Carina system (Drägerwerk AG&Co, Lübeck, Germany), H) standard diving regulator (Apeks Marine Equipment Ltd., Blackburn, United Kingdom). All devices are shown 3-5 and 8 are shown in Figure 1.

The devices 1-5 were used with constant flow oxygen at a rate of 15 L/min. This flow rate was chosen as 15 L/min can be achieved with most adjustable flow restrictors in prehospital and hospital settings. The nasal high-flow was set to 50 L/min flow, inspired oxygen fraction (FiO2) to 1.0 and temperature to 37.0°C. For non-invasive ventilation, PEEP was set to 12 mbar, ASB to 4 mbar and FiO2 to 1.0. The diving regulator was operated with pure oxygen supplied by a scuba tank and participants wore a nose clip when breathing with the regulator. A diving regulator works as a demand system, which enables the user to breathe comfortably by reducing a pressurized breathing gas to the ambient pressure. It provides neither pressure support nor PEEP and the flow rate depends on the breathing depth and frequency.

Each participant tested seven different oxygen systems in a randomized order. The devices 1, 2, 5 and 6 were used on all 30 participants. As the AirLife Open mask (device 3) became available for testing first during the course of the study, it was tested only on the last 14 participants. To not exceed the authorized number of seven devices per participant as determined by the ethics committee, the AirLife open mask replaced device 7 in 4 and devices 4 and 8 in each 5 participants.

### Selection of Participants

Participants of the study had to be non-smokers (former smokers allowed) aged 18–65 years. Exclusion criteria were clinically relevant cardiorespiratory diseases (for example COPD or asthma), former SARS-COV-2 infection, a body temperature >37.5°C, migraine, claustrophobia and coagulopathy/anticoagulant medication. Bearded volunteers were excluded if the beard impaired the fit of the face masks.

### Intervention

For the examination, participants were sitting upright in a comfortable chair in a quiet room. Participants were asked to breathe normally; through the nose in all nasal devices and through the nose or the mouth in those devices that cover both nose and mouth. Except for the speech break (see below), participants did not speak during the examination. Arterial access for serial blood gas analyses was obtained using a 20G cannula in the left or right radial artery. Blood gas analyses were performed on a Radiometer ABL90 Flex automated blood gas analyzer (Radiometer GmbH, Krefeld, Germany).

### Measurements

Blood samples were drawn at baseline and at 1, 2, 5 and 10 min after start of each oxygen system. To simulate a brief anamnesis, participants read a standardized text for 45 s after the 5-min blood draw. At the end of this speech break, another blood sample was drawn. As not only anamnesis but also possibility to drink may be important in (pre)clinical practice, the devices which allow speaking and drinking (nasal cannula, AirLife Open, SuperNO_2_VA, nasal high flow), remained in place, all devices which cover the mouth completely (non-rebreather mask, Flow-Safe II, NPPV, diving demand regulator) were removed during the 45-s speech break. A 15-min wash-out phase after each oxygen system allowed the participants to recover and pO2 to return to normal values. If the pO2 was still above 100 mm Hg after 15 min, the pause was extended until pO2 returned below 100 mm Hg. Body temperature was measured with a digital ear thermometer at the begin of each examination.

### Outcomes

Primary outcome was the arterial pO2 achieved with each system. Secondary outcomes were the influence on arterial pCO2 and pH and the subjectively perceived comfort reported by the participants.

To examine the subjective comfort/discomfort of the devices, participants were asked to grade each device in five categories: comfort, dyspnea, claustrophobia/anxiety, respiratory effort and dizziness. Grades ranged from one (excellent) to five (poor). At the end of the examination, participants had to order the devices according to their overall impression.

### Statistical Analysis

Data are shown as mean and standard deviation. All parameters were tested for normality by Shapiro-Wilk test. Differences in dependent variables were compared by paired *t*-test or Wilcoxon signed-rank test and differences in independent variables by unpaired *t*-test or Mann-Whitney *U*-test as appropriate. Pearson’s test or Spearman’s rank test were used for correlation analysis as appropriate. A two-sided *p*-value of less than 0.05 was considered statistically significant. All statistical analyses were carried out using the Statistical Package for the Social Sciences (SPSS) software package version 27.0.1.0 (IBM, USA).

## Results

### Demographics

The 17 female and 13 male participants were aged between 23 and 55 years (mean age 33.2 years, ± 8.8). Body mass indices (BMI) ranged from 18.6 to 30.0 kg/m^2^ (mean BMI 22.7 kg/m^2^, ±3.1) and body temperatures from 36.0 to 37.3°C (mean 36.6°C, ±0.3). Five participants were former smokers (1–36 pack years). Except arterial hypertension (n = 1) and rheumatoid arthritis (n = 1) participants reported no comorbidities.

### Oxygenation

**FIGURE 2:**
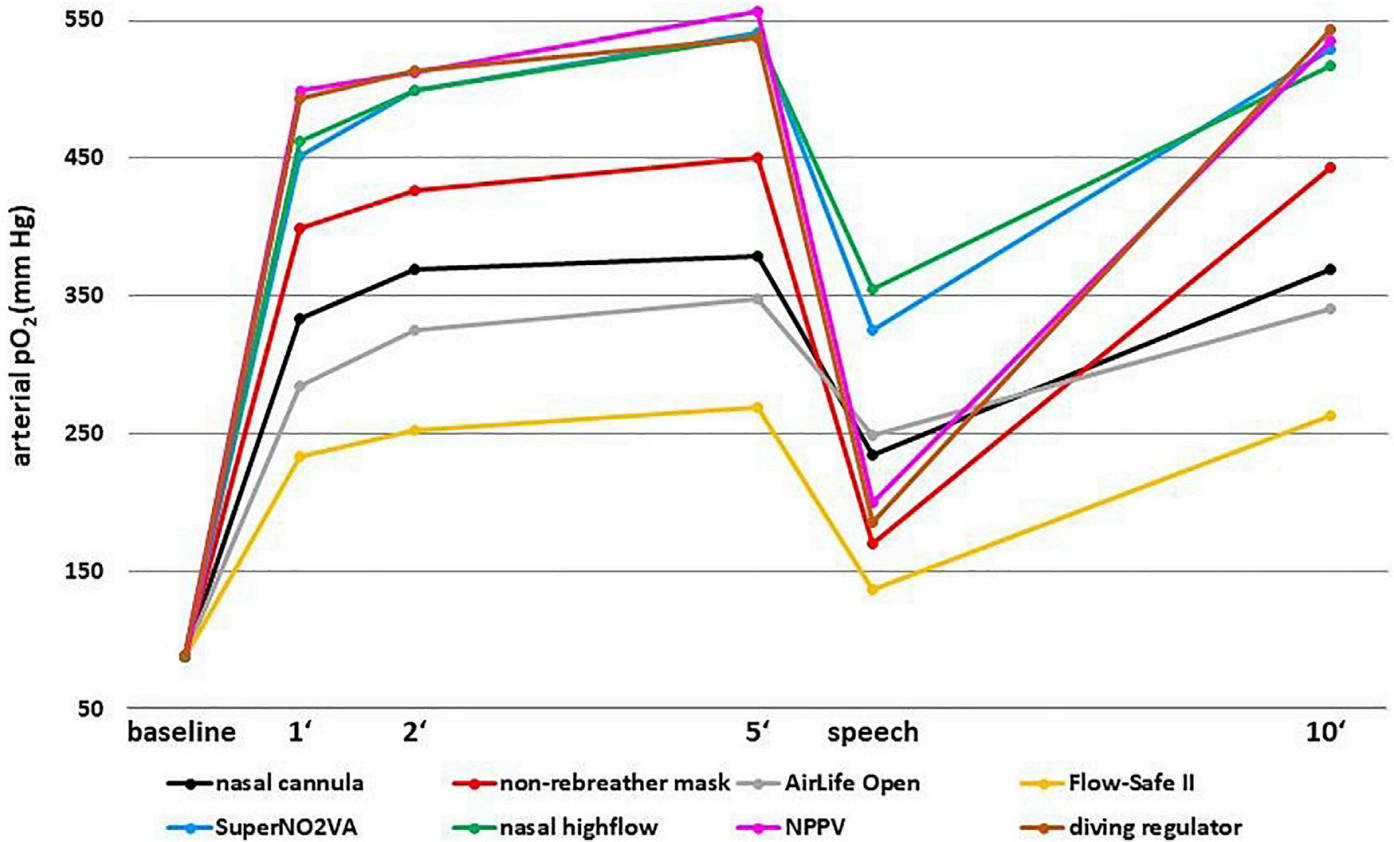
Mean arterial pO2 (mm Hg) achieved with the different oxygen systems.

All oxygen systems significantly increased arterial pO2 within the first minute (all *p* < 0.001) and a maximum plateau was achieved at the 5-min time point (Figure 2 and Table 1). Although the increase between minute one and five was also statistically significant in all devices (all *p* < 0.001), the slope of this increase was much less pronounced compared to the initial increase until minute one. At minute one, the mean arterial pO2 was between 83% (AirLife Open) and 92% (diving regulator) of the pO2 achieved at minute five (SuperNO_2_VA: 84%, nasal high-flow: 86%, Flow-Safe II: 87%, nasal cannula and non-rebreather mask: 89% and NPPV: 90%).

**TABLE 1:** Mean arterial pO2 (mm Hg) achieved with the different oxygen systems (mean and standard deviation).

	Baseline	1′	2′	5′	Speech	10′
Nasal cannula (n = 30)	89 (±7)	334 (±55)	370 (±57)	379 (±62)	235 (±46)	370 (±72)
Non-rebreather mask (n = 30)	88 (±7)	399 (±46)	427 (±42)	451 (±52)	171 (±25)	443 (±50)
AirLife Open (n = 14)	90 (±5)	285 (±30)	325 (±39)	348 (±52)	249 (±35)	341 (±45)
Flow-Safe II (n = 25)	88 (±7)	234 (±44)	253 (±46)	270 (±51)	137 (±20)	264 (±52)
SuperNO_2_VA (n = 30)	89 (±9)	452 (±42)	499 (±38)	541 (±28)	325 (±75)	529 (±30)
Nasal high-flow (n = 30)	89 (±7)	463 (±52)	500 (±47)	539 (±47)	355 (±64)	517 (±47)
NPPV (n = 26)	90 (±9)	499 (±48)	513 (±30)	556 (±28)	200 (±36)	535 (±44)
Diving regulator (n = 25)	90 (±5)	494 (±49)	514 (±41)	538 (±36)	186 (±36)	544 (±36)

SuperNO_2_VA, NPPV, nasal high-flow and diving regulator were the most effective devices achieving mean arterial pO2 concentrations well above 500 mm Hg. The AirLife Open mask, the nasal cannula and the non-rebreather mask achieved intermediate concentrations of about 350–450 mm Hg. The least effective system was the Flow-Safe II device, which achieved a mean arterial pO2 of 270 mm Hg at 5 minutes. The pressure values displayed by the Flow-Safe II manometer ranged from 9 to 14 cm H_2_0 (mean 11 cm H_2_0, ±1.1). PO2 at minute ten but not at any other time point correlated significantly with the displayed pressure (r = 0.418, *p* = 0.038).

The highest variability between participants was observed with the nasal cannula, where 5-min measurements ranged from 286 to 526 mm Hg. The highest pO2 measured in this study was 607 mm Hg achieved with the nasal high-flow at 5 min.

The 45-s speech break caused a significant drop in arterial pO2 in all oxygen systems (all *p* < 0.001). This drop was less pronounced with the devices which remained in place during speaking (% drop from 5-min measurement: AirLife Open 27%, nasal high-flow 34%, nasal cannula 38%, SuperNO_2_VA 40%), compared to those devices which had to be removed completely (Flow-Safe II 49%, non-rebreather mask 62%, NPPV 64%, diving regulator 65%). At the 10-min measurement, the arterial pO2 had returned to the maximum plateau, which was comparable to that measured at minute five.

There was a weak negative correlation between baseline pO2 (before first oxygen application) and age, which just did not reach statistical significance (r = - 0.362, *p* = 0.050). Except a negative correlation between pO2 and age at 5 minutes with the nasal high-flow (r = -0.510, *p* = 0.004), pO2 concentrations at minute five and ten did not correlate with age in all other devices. There was no significant difference in baseline pO2 between men and women and no consistent differences between sexes in pO2 at five and 10 minutes.

### Ventilation

**FIGURE 3:**
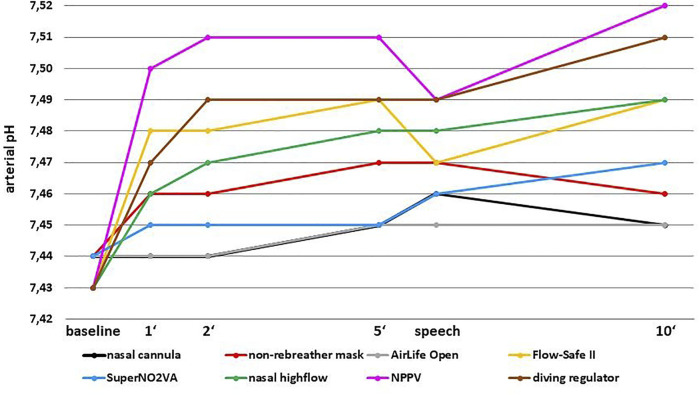
Mean arterial pH during breathing with the different oxygen systems.

Participants tended to hyperventilate with all devices except for the AirLife Open mask (Tables 2, 3, Figure 3). This effect was very mild and transient with the nasal cannula as the increase in pH and pCO2 was statistically significant only at minute five (*p* = 0.050 for pH and 0.027 for pCO2) but not anymore at minute ten. For the SuperNO_2_VA, the increase in pH at minute five was just not statistically significant (*p* = 0.055) but became significant at minute ten (*p* < 0.001). With all the remaining devices, the increase in mean pH and mean pCO2 from baseline to minute five and ten was statistically significant (all *p* < 0.002). The highest pH values were observed with NPPV, diving regulator, Flow-Safe II and nasal high-flow. Except a positive correlation between pO2 and pCO2 (r = 0.499, *p* = 0.005) for the non-rebreather mask at minute five, no further correlations between pCO2 and pO2 at five or 10 minutes occurred.

**TABLE 2:** Mean arterial pCO2 (mm Hg) during breathing with the different oxygen systems (mean and standard deviation).

	Baseline	1′	2′	5′	Speech	10′
Nasal cannula (n = 30)	36 (±3)	35 (±5)	35 (±5)	34 (±4)	33 (±4)	35 (±4)
Non-rebreather mask (n = 30)	36 (±4)	34 (±5)	33 (±5)	32 (±5)	32 (±4)	33 (±5)
AirLife Open (n = 14)	35 (±3)	35 (±4)	34 (±4)	34 (±4)	33 (±4)	34 (±4)
Flow-Safe II (n = 25)	37 (±3)	31 (±5)	30 (±5)	30 (±6)	32 (±4)	30 (±7)
SuperNO_2_VA (n = 30)	36 (±4)	34 (±5)	34 (±5)	34 (±5)	33 (±4)	33 (±5)
Nasal high-flow (n = 30)	36 (±3)	32 (±4)	32 (±4)	31 (±5)	31 (±4)	30 (±6)
NPPV (n = 26)	36 (±3)	29 (±4)	28 (±4)	28 (±5)	30 (±3)	27 (±5)
Diving regulator (n = 25)	36 (±3)	32 (±4)	31 (±4)	30 (±5)	31 (±4)	29 (±5)

**TABLE 3:** Mean arterial pH during breathing with the different oxygen systems (mean and standard deviation).

	Baseline	1′	2′	5′	Speech	10′
Nasal cannula (n = 30)	7.44 (±0.03)	7.44 (±0.04)	7.44 (±0.04)	7.45 (±0.03)	7.46 (±0.04)	7.45 (±0.04)
Non-rebreather mask (n = 30)	7.44 (±0.03)	7.46 (±0.04)	7.46 (±0.05)	7.47 (±0.05)	7.47 (±0.04)	7.46 (±0.05)
AirLife Open (n = 14)	7.44 (±0.02)	7.44 (±0.03)	7.44 (±0.03)	7.45 (±0.03)	7.45 (±0.03)	7.45 (±0.04)
Flow-Safe II (n = 25)	7.43 (±0.03)	7.48 (±0.04)	7.48 (±0.04)	7.49 (±0.05)	7.47 (±0.03)	7.49 (±0.07)
SuperNO_2_VA (n = 30)	7.44 (±0.03)	7.45 (±0.04)	7.45 (±0.04)	7.45 (±0.04)	7.46 (±0.03)	7.47 (±0.05)
Nasal high-flow (n = 30)	7.43 (±0.03)	7.46 (±0.04)	7.47 (±0.04)	7.48 (±0.05)	7.48 (±0.04)	7.49 (±0.06)
NPPV (n = 26)	7.43 (±0.03)	7.50 (±0.03)	7.51 (±0.04)	7.51 (±0.05)	7.49 (±0.04)	7.52 (±0.05)
Diving regulator (n = 25)	7.43 (±0.03)	7.47 (±0.05)	7.49 (±0.05)	7.49 (±0.06)	7.49 (±0.04)	7.51 (±0.06)

Although hyperventilation was statistically significant, it was not clinically apparent except for the diving regulator. There was a significant negative correlation between dizziness and pCO2 and a positive correlation between dizziness and pH, both at minute five (pCO2: r = -0.515, *p* = 0.008; pH: r = 0.492, *p* = 0.012) and ten (pCO2: r = -0.508, *p* = 0.009; pH: r = 0.502, *p* = 0.011). No such correlation was found for any other device.

### Comfort/Discomfort

Participants were asked to grade each device in five categories, where one was the best and five the poorest possible grade (Table 4). In terms of comfort, the AirLife Open mask (mean grade 1.1) and the non-rebreather mask (1.3) performed best, whereas SuperNO_2_VA (3.2) and nasal cannula (3.5) performed worst. Subjectively perceived dyspnea was generally mild but more pronounced in those devices that generate a positive airway pressure (NPPV: 1.8, SuperNO_2_VA: 1.8, Flow-Safe II: 1.5). The feeling of anxiety was especially present in those devices that work with tight fitting masks (NPPV: 2.0, SuperNO_2_VA: 2.0, Flow-Safe II: 1.9). Similar to the feeling of dyspnea, the effort of breathing was worst in positive airway pressure generating devices (SuperNO_2_VA: 2.9, NPPV: 2.8, Flow-Safe II: 2.2). More distinct dizziness was uncommon in all devices where mean grades ranged from 1.1 (non-rebreather mask) to 1.7 (Flow-Safe II). Besides the discomfort that was reported for some systems, no complications such as epistaxis or irritability of the airway occurred.

**TABLE 4:** Subjective comfort ratings (mean, standard deviation) as reported by the participants. Grading ranged from 1 (excellent) to 5 (poor).

	Comfort	Dyspnea	Anxiety	Effort	Dizziness
Nasal cannula (n = 30)	3.5 (±1.1)	1.1 (±0.3)	1.1 (±0.3)	1.1 (±0.3)	1.4 (±0.6)
Non-rebreather mask (n = 30)	1.3 (±0.5)	1.0 (±0.2)	1.1 (±0.3)	1.1 (±0.3)	1.1 (±0.3)
AirLife Open (n = 14)	1.1 (±0.4)	1.1 (±0.3)	1.1 (±0.4)	1.1 (±0.3)	1.2 (±0.6)
Flow-Safe II (n = 25)	2.5 (±0.7)	1.5 (±0.7)	1.9 (±1.1)	2.2 (±1.0)	1.7 (±0.8)
SuperNO_2_VA (n = 30)	3.2 (±1.0)	1.8 (±1.0)	2.0 (±1.0)	2.9 (±1.1)	1.4 (±0.7)
Nasal high-flow (n = 30)	1.7 (±0.7)	1.2 (±0.4)	1.3 (±0.6)	1.6 (±0.7)	1.4 (±0.7)
NPPV (n = 26)	2.9 (±1.1)	1.8 (±0.8)	2.0 (±1.0)	2.8 (±1.1)	1.4 (±0.7)
Diving regulator (n = 25)	2.4 (±1.0)	1.4 (±0.6)	1.6 (±0.8)	1.8 (±0.9)	1.4 (±0.8)

The non-rebreather and the AirLife Open masks showed an exceptional small variance between participants (only grades 1 and 2 in all categories except one grade 3 in the comfort category for the non-rebreather mask and one grade 3 for dizziness for the AirLife Open). The other devices showed considerable variance in some categories as illustrated by the standard deviations in Table 4.

When participants were asked for their overall impression, the devices were ranked as follows (average rank): 1. AirLife Open (1.9), 2. non-rebreather mask (2.0), 3. nasal high-flow (2.7), 4. diving regulator (3.9), 5. Flow-Safe II (4.4), 6. NPPV (5.0), 6. SuperNO_2_VA (5.5) and 8. nasal cannula (5.8).

## Discussion

HBO treatment is the gold standard of treatment for patients with DCI and severe smoke and CO intoxication (Weaver, 1999; Ortega et al., 2021). With increasing arterial pO2, inert gas bubbles dissolve and O2 replaces CO in heme-containing enzymes. Furthermore, the increase of physically dissolved oxygen in the blood improves tissue oxygenation (Weaver, 1999; Ortega et al., 2021). As HBO treatment is only available in specialized centres, arterial pO2 must be increased as much as possible using normobaric modalities during first aid, primary and secondary transport (Eichhorn et al., 2018; Mitchell et al., 2018a; Mitchell et al., 2018b; Juttner et al., 2021). In this study, we investigated eight oxygen systems for their ability to increase arterial pO2 and their comfort. Beside common systems, we also included the SuperNO_2_VA nasal PAP device and the Flow-Safe II CPAP device as they are used with constant low flow oxygen, do not need power supply or highly skilled providers and are therefore particularly suitable for a prehospital setting.

A standard diving regulator operated with pure oxygen and the SuperNO_2_VA mask used with constant flow oxygen at a rate of 15 L/min achieved comparable arterial pO2 as nasal high-flow and the gold standard NPPV. The SuperNO_2_VA is a nasal PAP system that was initially developed to stent open the upper airway in a preoperative setting. Mean arterial pO2 was >500 mm Hg with these devices and the increase of pO2 was rapid with near maximum values reached within 1 minute. The other systems, including a standard nasal cannula and a standard non-rebreather mask, were less effective. According to the alveolar gas equation, the maximal theoretically attainable arterial pO2 under 100% normobaric oxygen is about 660 mm Hg at sea level and about 612 mm Hg at our localisation, which is 570 m above sea level. Due to the alveolar-arterial drop, physiological shunts, V/Q ratio, exhaled pN2 and pCO2, the pO2 values realizable in practice are about 500–550 mmHg. Therefore, the oxygenation performance of the best devices in our study was within the theoretically achievable maximum and even endotracheal intubation would not improve these results.

The good oxygenation performance of the diving regulator and the SuperNO_2_VA nasal PAP device has implications on the primary care of patients with DCI or smoke and CO intoxication. At many diving sites, scuba tanks with pure oxygen are available. In case of DCI, it may be more effective to use a standard diving regulator operated with pure oxygen than a nasal cannula or non-rebreather mask, which are usually applied by first responders for primary care and transport to hospital. NPPV and nasal high-flow devices are limited for primary care and transport as devices are complex requiring a trained physician or the devices are not portable without constant power supply. Hence, the SuperNO_2_VA, which is easy to handle and operated with constant flow from a standard oxygen tank, could be an equally effective alternative to NPPV or nasal high-flow for primary and secondary transports providing maximal oxygenation.

The least effective device for increasing arterial pO2 was the Flow-Safe II system, which is a disposable CPAP system that generates a PEEP of approximately 11–12 cm H_2_O at a flow rate of 15 L/min. This finding is surprising, as the Flow-Safe II device has been shown to improve blood gas parameters in patients with acute cardiogenic pulmonary edema as effective as NPPV (Uz et al., 2021). An explanation for the lower efficacy of the Flow-Safe II system in our study may be that we included only healthy volunteers without cardiopulmonary disease. While in patients with pulmonary edema or other acute pulmonary diseases the application of PEEP significantly improves oxygenation, PEEP is less important than FiO2 for oxygenation in non-obese persons without pulmonary pathology (Pelosi et al., 1999; Coruh and Luks, 2014). FiO2 delivery of the Flow-Safe II device decreases with increasing tidal volume and respiratory rate. As hyperventilation occurred with the Flow-Safe II device, FiO2 may have decreased markedly below 1.0 contributing to the unexpected low oxygenation performance.

In case of CO intoxication, a lesser oxygenation efficacy translates into a longer CO dissociation half-life, which may promote tissue damage. The four most effective devices (NPPV, nasal high-flow, diving regulator and SuperNO_2_VA) achieved a mean arterial pO2 of about 540 mm Hg, which was twofold the mean pO2 achievable with the least effective device. This difference would result in a clinically significant reduction of CO dissociation half-life from estimated 200 min with the least effective device to about 74 min with the most effective devices (Rose et al., 2017). A further reduction of carboxyhemoglobin half-life can only be obtained with HBO therapy. A recent study in patients with CO intoxication supports this assumption as oxygen therapy via nasal high-flow lead to a faster reduction of carboxyhemoglobin compared to oxygen therapy via conventional face mask (Tomruk et al., 2019).

We did not only investigate oxygenation performance, but also if the different oxygen systems were associated with hyperventilation. Hyperventilation can result in higher arterial pO2 by increasing the alveolar fiO2 fraction, but might be disadvantageous in case of DCI due to increased oxygen consumption and decreased peripheral tissue perfusion (de Villalobos et al., 2021; Zhou et al., 2021). Except for the AirLife Open, mild hyperventilation was observed with all oxygen systems with maximal pH values of about 7.5. Interestingly, the only device where hyperventilation was clinically apparent as indicated by a correlation between pH, pCO2 and dizziness reported by the participants, was the diving regulator. This finding may be biased by the fact that most participants had never used a diving regulator before and reported that breathing with the regulator was very unfamiliar. As DCI occurs in persons who are used to breathing with a diving regulator, hyperventilation may not be present in a diver cohort. Also in terms of hyperventilation, the SuperNO_2_VA performed well with lower pH values at all time points compared to the diving regulator, NPPV and nasal high-flow.

Aside from pure efficacy, patient comfort and practicability are further important factors for oxygen systems used in an emergency setting. Although all devices tested were tolerated for 15 min without any premature discontinuation, subjective reported comfort differed between the individual systems. To our surprise, the nasal cannula was rated the least comfortable system followed by the SuperNO_2_VA. At a flow of 15 L/min oxygen, the nasal cannula was described as burning and painful. In contrast, the Air Life Open mask and the standard non-rebreather mask were perceived as very comfortable by mostly all participants. Dyspnea and the effort of breathing were more pronounced in devices that work with a positive airway pressure, anxiety was more common in tight fitting mask systems. Given the experience from this study, we expect that in an emergency it should be possible to bridge a timeframe of one to 2 hours until a HBO center can be reached with all devices.

Practicability is another important aspect especially in the pre-hospital setting and during transport. As already discussed above, NPPV and nasal high-flow devices need a power supply and specifically trained staff. In contrast, all other devices only need low flow oxygen supply via a standard connector and can be handled easily also from staff without special knowledge.

Beside maximal oxygenation, early and proper hydration is another mainstay in the acute treatment of DCI (Mitchell et al., 2018a; Mitchell et al., 2018b). In addition, detailed anamnesis is crucial to distinguish between different forms of DCI. By not covering the mouth, the nasal cannula, nasal high-flow, Air Life open and SuperNO_2_VA enable patients to speak and drink without removing the device. This can be advantageous compared to the full-face masks used for the Flow-Safe II device and NPPV and the non-rebreather mask and diving regulator. When these devices are removed for speech or drinking breaks, arterial pO2 rapidly and significantly decreases. This emphasizes the importance of minimizing all interruptions, keeping patient interviews short and helping patients to focus on proper breathing. Proper breathing is especially important in all nasal devices as a decrease in arterial pO2 also occurs in these devices when patients do not breathe through the nose. Therefore, nasal devices might be more suited as bridging devices and less for longer treatment episodes when patients would have to concentrate not to breathe through the mouth.

The main limitation of this study is that only healthy individuals were included. Therefore, the results of this study cannot be transferred to patients with impaired oxygenation, as for example cardiogenic pulmonary edema or (viral) pneumonia. As already discussed, further parameters such as PEEP may become more important in those patients. From our first experience in patients with COVID-19 pneumonia (data not shown) and as reported in patients with pulmonary edema (Uz et al., 2021), devices like the Flow-Safe II that generate PEEP may perform better in those cardiopulmonary compromised patients than they did in our healthy subjects. As most divers and many victims of smoke and CO intoxication normally do not suffer from severe cardiopulmonary disease that impairs oxygenation, we are therefore confident that the conclusions drawn from of this study can be applied to this special patient cohort.

In conclusion, our study confirms a high oxygenation performance of several non-invasive oxygen devices in cardiopulmonary healthy subjects with arterial pO2 values reaching the theoretically achievable maximum. Not only nasal high-flow and NPPV but also a standard diving regulator operated with pure oxygen and the SuperNO_2_VA device used with constant flow oxygen were more effective in increasing arterial pO2 compared to a standard nasal cannula and a non-rebreather mask. They may be valuable alternatives for primary care and transport of patients with smoke and CO intoxication and decompression illness.

## Data Availability

The original contributions presented in the study are included in the article/Supplementary Material, further inquiries can be directed to the corresponding author.
